# Panton-Valentine Leukocidin–Encoding Methicillin-Resistant *Staphylococcus aureus,* the Netherlands, 2023–2024^1^

**DOI:** 10.3201/eid3204.251646

**Published:** 2026-04

**Authors:** Patrick van Schelven, Roel Nijhuis, Casper Jamin, Sophie Goemans, Putri Hintaran, Marischka van der Jagt-Zwetsloot, Annelies Smilde, Annelot ter Horst, Melanie de Graaf, Daan W. Notermans, Anne Russcher, Stijn Raven

**Affiliations:** Public Health Service Region Utrecht, Zeist, the Netherlands (P. van Schelven, S. Goemans, P. Hintaran, M. van der Jagt-Zwetsloot, A. ter Horst, S. Raven); Meander Medical Centre, Amersfoort, the Netherlands (R. Nijhuis, A. Smilde, A. Russcher); National Institute for Public Health and the Environment, Bilthoven, the Netherlands (C. Jamin, D.W. Notermans); Unilabs-Saltro Diagnostic Centre, Utrecht, the Netherlands (M. de Graaf)

**Keywords:** methicillin-resistant *Staphylococcus aureus*, MRSA, bacteria, antimicrobial resistance, disease outbreak, public health, the Netherlands

## Abstract

We describe a community outbreak of Panton-Valentine leukocidin–positive methicillin-resistant *Staphylococcus aureus* (MRSA) during November 2023–June 2024 in the Netherlands. We identified a massage center as the source. Case-patients experienced skin infections and abscesses. This study highlights the importance of genomic surveillance of MRSA in distinguishing Panton-Valentine leukocidin–positive MRSA.

Methicillin-resistant *Staphylococcus aureus* (MRSA) of multilocus sequence type (MLST) clonal complex (CC) 398 is usually considered livestock-associated (LA) MRSA. Panton-Valentine leukocidin (PVL)–positive isolates of this type are increasingly detected in humans and not associated with livestock exposure (*1*–*5*). Those strains possess virulence genes that enhance human-to-human transmission (*6*). In November 2023, an outbreak of PVL-positive CC398 MRSA linked to a massage center occurred in the Netherlands. Here, we describe the course and characteristics of the outbreak and the management challenges specific to a community MRSA outbreak.

## The Study

In November 2023, a microbiologic hospital laboratory notified the regional Public Health Service (PHS) of a potential outbreak of PVL-positive CC398 MRSA in the region. Within 3 weeks, 5 patients sought care at the hospital with MRSA-positive abscesses that required surgical drainage. All isolates were multilocus variable-number tandem-repeat analysis (MLVA) type MT2306, MLVA complex MC0398, a previously rarely encountered MLVA type. After notification, the PHS interviewed reported patients to identify potential infection sources and contacts at risk and collected clinical isolates for enhanced genomic surveillance using whole-genome sequencing (WGS) to further distinguish identical MLVA types.

**Figure 1:**
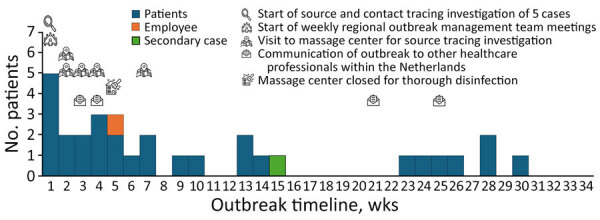
Epicurve and timeline of outbreak of Panton-Valentine leukocidin–encoding methicillin-resistant *Staphylococcus aureus*, the Netherlands, 2023–2024. Timeline of the outbreak in weeks was based on the date of notification to the Public Health Service, with the actions undertaken during the outbreak.

From the first 5 interviews, we identified a specific massage center as a potential source location. PHS staff visited the massage center on 5 occasions during the outbreak (Figure 1). Employees of the center were screened for MRSA at multiple time points (Appendix Table 1). Initially, all employee samples tested negative; at the fourth visit, an employee previously unknown to the PHS was present and tested positive. The employee of the massage center who tested positive was advised to refrain from work and undergo MRSA eradication therapy. The employee resumed work after 3 consecutive negative tests. The employee reported no risk factors for MRSA acquisition (e.g., travel history). We conducted environmental sampling on 3 occasions; multiple samples tested positive (Appendix Table 1).

We identified a total of 31 cases during November 2023–June 2024 (Figure 1). Median age of all case-patients was 42 (range 3–69) years, and all but 1 were >18 years of age; 42% were female and 58% male. Of the 31 case-patients, 27 were symptomatic, 3 asymptomatic, and 1 of unknown status. Immune status of 3 case-patients was unknown, and none of the other case-patients were immunocompromised; 1 had type 2 diabetes mellitus. Most case-patients had mild skin infections from the MRSA infection. Ten cases required hospitalization; of those, 8 case-patients underwent surgery to relieve abscesses and 1 was admitted to the hospital with bacteremia.

In total, 22 of 31 case-patients reported visiting the massage center. Six case-patients did not report visiting the massage center; of those 6 cases, 1 was a secondary case because the patient was a household contact. The remaining 3 case-patients did not cooperate in source and contact investigation; nevertheless, we included them in the outbreak because of their geographic and microbiologic links. A year after the last notification, no new MRSA isolates of this specific MC0398-MT2306 strain had been reported in the PHS region, according to national MRSA surveillance.

We tested for the *mecA/mecC* gene in *S. aureus*–positive cultures using a laboratory-developed real-time PCR. We used an enrichment method to culture nose, throat, and rectal swab specimens (*7*). During the outbreak, we obtained environmental specimens in 2 ways: by wiping surfaces using gauze moistened with sterilized water and inserting the gauze in overnight enrichment broth, or by using PL agar contact plates (AnalytiChem, https://www.analytichem.com) and incubating them at 35°C for 24 hours. We took all colony-forming units (CFU) from the contact plates and inserted them in overnight enrichment broth, then tested the enrichment broths and CFU from the contact plates.

**Figure 2:**
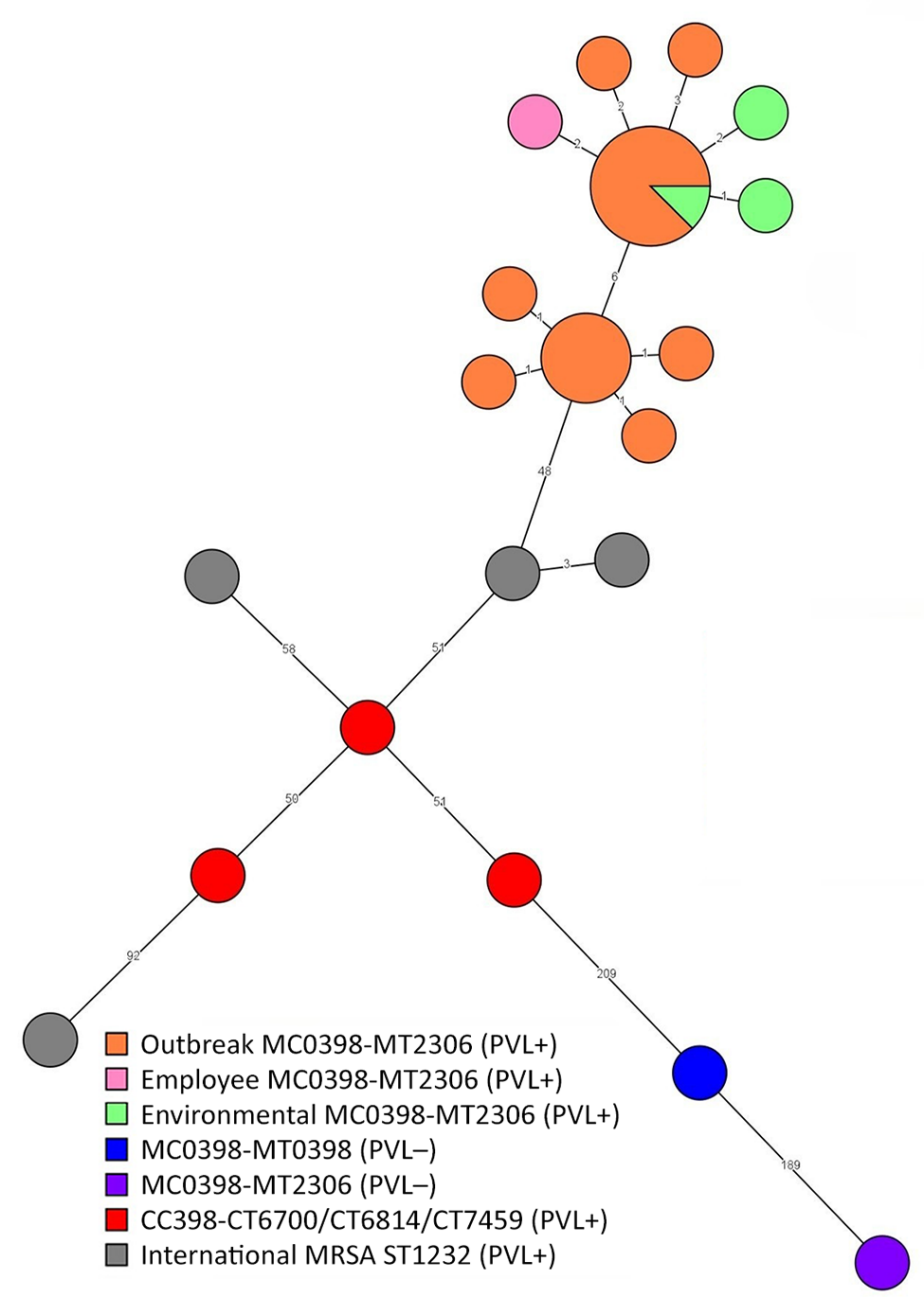
Whole-genome multilocus sequence typing minimum spanning tree in study of outbreak of PVL-encoding MRSA, the Netherlands, 2023–2024. We determined that all MRSA isolates were MLVA type MT2306, complex MC0398, similar to CC398. Tree shows the outbreak isolates of human cases (orange), employee case (pink), and environmental samples (green) clustering with a maximum difference of 12 alleles, confirming a microbiological link between the patients and the environmental samples. National PVL-positive, CC398 MRSA strains (red) and international PVL-positive MRSA ST1232 strains (gray) differ >48 alleles compared with the outbreak strain. The genome from a PVL-negative MC0398-MT2306 (purple) isolate from 2019 differs on 238 alleles from the outbreak isolates; the genome of the PVL-negative MC0398-MT0398 (blue) isolate differs 226 alleles from the outbreak isolates. CC, clonal complex; MLVA, multilocus variable number tandem repeat analysis; MRSA, methicillin-resistant *Staphylococcus aureus;* PVL, Panton-Valentine leucocidin; ST, sequence type.

We determined that all MRSA isolates in the outbreak were MLVA type MT2306, complex MC0398, similar to CC398, as described previously (*8*). National surveillance data showed that this type was previously found in 4 isolates sampled during 2010–2021 in a different region of the country. All 4 previous isolates had tested PVL-negative. We performed WGS on MRSA isolates from the first 17 cases, the employee and all environmental isolates, as described previously (*9*). All MRSA isolates of the MC0398-MT2306 cluster belonged to MLST type ST1232 and Ridom core genome MLST complex type 35023 (Ridom GmbH, https://www.ridom.de). We analyzed genomic relatedness by whole-genome MLST analysis, using a cutoff of <15 alleles difference (*8*). The range of different alleles among all isolates related to the outbreak was 0–12 (mean 1) alleles. The 3 environmental isolates clustered with the human isolates with <1 allele difference. We compared the genomes from the 17 human isolates in our study with genomes of a PVL-negative MC0398-MT2306 strain isolated in 2019 that originated from a person who had close contact with pigs; that strain was ST398, complex type 7121, a PVL-negative MC0398-MT0398 from November 2023. We also compared those genomes with 4 international PVL-positive MRSA ST1232 isolates from the National Center for Biotechnology Information RefSeq database (https://www.ncbi.nlm.nih.gov/refseq; accession nos. GCF_019669165, GCF_019669245, GCF_019669345, GCF_019669385). The MC0398-MT2306 PVL-negative isolate from 2019 differed by 238 alleles from the nearest PVL-positive CC398 MRSA from the cluster in our outbreak, the MC0398-MT0398 PVL-negative differed by 226 alleles to isolates from our cluster, and the international PVL-positive MRSA ST1232 isolates differed by >48 alleles from our cluster (Figure 2). We also examined antimicrobial resistance in the study isolates (Appendix Tables 2, 3).

After notification of the first 5 cases, the region formed an outbreak management team composed of physicians and nurses of the PHS, medical microbiologists of the notifying laboratory, infection prevention experts of both the PHS and the hospital, and communication specialists. All general practitioners in the area were notified and advised to culture infected skin lesions not responding to first-line antimicrobial treatment. The outbreak was communicated via the national surveillance report 21 days after notification to alert other PHS and clinical microbiologists. In addition, the National Institute for Public Health and the Environment issued an alert via EpiPulse, an online platform for sharing infectious disease among public health authorities in Europe.

We interviewed case-patients to identify potential at-risk contacts. We requested that symptomatic contacts, asymptomatic household contacts, and contacts working in healthcare or having a profession with intensive physical contact (i.e., masseuse, skin therapist) undergo testing for MRSA. It was not possible to screen all clients of the source location because of privacy issues. We advised all case-patients to undergo MRSA eradication therapy after full lesion healing, to prevent reintroduction in the source location. An infection prevention expert advised the staff at the source location about MRSA transmission and hygiene measures. In addition, the source location was thoroughly disinfected during the outbreak by a professional disinfection company in week 5 of the outbreak. The expert assisted by developing a disinfection plan, explaining its necessity and addressing questions of the source location’s staff. The expert advised that objects that could not be sufficiently disinfected (e.g., fabric-covered tissue boxes, fabric decoration pieces) be permanently removed.

## Conclusions

We report a PVL-positive MRSA ST1232 strain similar to CC398 that caused a community outbreak with severe skin infections in the Netherlands. We confirmed human-to-human transmission in the community and detected viable MRSA on surfaces at the source location. This outbreak underscores the rise of PVL-positive CC398 MRSA strains in the community and its outbreak potential. The community setting of the source location posed several challenges in outbreak management, emphasizing the need for a tailored approach and rapid source tracing.

Appendix Additional information about outbreak of Panton-Valentine leukocidin–encoding CC398-like methicillin-resistant *Staphylococcus aureus*, the Netherlands, 2023–2024
