# Supplementary material for: Panton-Valentine Leukocidin–Encoding Methicillin-Resistant Staphylococcus aureus, the Netherlands, 2023–2024
Source: Emerg Infect Dis. 2026 Apr;32(4):640–3. doi: 10.3201/eid3204.251646 (PMC13094855; doi:10.3201/eid3204.251646)
Supplement: Appendix — Additional information about outbreak of Panton-Valentine leukocidin–encoding CC398-like methicillin-resistant Staphylococcus aureus, the Netherlands, 2023–2024 [file 25-1646-Techapp-s1.pdf]

Article DOI: https://doi.org/10.3201/eid3204.251646

*EID cannot ensure accessibility for supplementary materials supplied by authors. Readers who have difficulty accessing supplementary content should contact the authors for assistance.*

# Panton-Valentine Leukocidin–Encoding Methicillin-Resistant *Staphylococcus aureus,* the Netherlands, 2023–2024

## Appendix

**Appendix Table 1.** Overview of samples collected at the massage center from employees and environmental sources*

| Sample | First visit (t = 7 d) | Second visit (t = 12 d) | Third visit (t = 20 d) | Fourth visit (t = 27 d) | Fifth visit (after disinfection) (t = 36 d) |
|---|---|---|---|---|---|
| Employee samples | | | | | |
| Employee 1 | Negative | | Negative | Negative | |
| Employee 2 | Negative | | Negative | Negative | |
| Employee 3 | Ns† | | Ns† | Positive (nose/throat/skin); Negative (rectum) | |
| Environmental samples | | | | | |
| Bed sheets | | Negative | | | |
| Hand alcohol bottle | | Negative | | | |
| Lamp above bed | | Negative | | | |
| Massage bench towel | | Negative | | | |
| Massage lotion | | Negative | | | |
| Massage oil | | Negative | | | |
| Massage oil cannister | | Negative | | | |
| Tiger balm | | Negative | | | |
| Toilet sink | | Negative | | | |
| Wooden frame bed | | Negative | | | |
| Working table | | Negative | | | |
| Curtain | | | | Negative | |
| Feet bench | | | | Negative | |
| Massage bench 1 | | | | Negative | |
| Massage bench 2 | | | | Negative | |
| Mattress (1) | | | | Negative | |
| Mattress (2) | | | | Negative | |
| Pedicure plastic tray | | | | Negative | |
| Peppermint jar | | | | Negative | |
| Scissor and plasticized desk card | | | | Negative | |
| Scrub salt | | | | Negative | |
| Shoehorn | | | | Negative‡ | |
| Sitting stool | | | | Negative | |
| Phone | | | | Negative‡ | Negative‡ |
| Tissue box (fabric) | | | | Positive‡ | Ns§ |
| Waste bin handle | | | | Positive‡ | Negative‡ |
| Water cooker | | | | Positive‡ | Negative‡ |
| Comb | | | | | Negative‡ |
| Fan | | | | | Negative‡ |
| Laptop | | | | | Negative‡ |
| Tea box | | | | | Negative‡ |
| Toothbrush | | | | | Negative‡ |

*Overview of samples collected at the massage center from employees and environmental sources. During the first visit, employees performed self-sampling of nasal and throat swabs. During subsequent visits, the infection prevention expert collected nasal, throat, and rectal swabs from the employees. Environmental samples were selected based on the infection prevention expert's assessment and comprised materials with direct skin contact involving employees, clients, or both. Samples were taken using gauze moistened with sterilized water or by PL agar contact plate.
†Ns = not screened (the third employee was unknown to the Public Health Service until day 27 and therefore was not screened earlier).
‡Sample collected using a PL agar contact plate.
§Ns = not screened (object was discarded during disinfection on day 28 and 29 and therefore unavailable for re-testing).

**Appendix Table 2.** MIC and interpretation of phenotypical resistance for antimicrobials*

| Antimicrobial | MIC range (mg/L) | Interpretation according to EUCAST Clinical Breakpoint Tables† |
|---|---|---|
| Oxacillin | >2 - ≥4 | R |
| Ciprofloxacin | ≤0.5 | I |
| Levofloxacin | 0.25 – 0.5 | I |
| Gentamicin | >8 - ≥16 | R |
| Tobramycin | 8 - ≥16 | R |
| Teicoplanin | ≤0.5 | S |
| Vancomycin | ≤0.5 – 1 | S |
| Clindamycin | >2 - ≥4 | R |
| Erytromycin | >4 - ≥8 | R |
| Tetracycline | >8 - ≥16 | R |
| Linezolid | 1 - 2 | S |
| Co-trimoxazole | 80 - ≥320 | R/I |
| Fusidic acid | ≤0.5 | S |
| Rifampin | ≤0.03 | S |
| Trimethoprim | | R‡ |
| Mupirocin | ≤1.0 | S |

*Minimal inhibitory concentration (MIC) of various antibiotics as determined by VITEK (Biomerieux) and the phenotypic resistance interpretation, based on EUCAST breakpoint values of the MIC during the testing period (2023 and 2024). S = susceptible, I = susceptible, increased exposure, R = resistant.
†European Committee on Antimicrobial Susceptibility Testing (EUCAST). EUCAST clinical breakpoints. *https://www.eucast.org/*. Accessed January 28, 2026.
‡Susceptibility tested using disk diffusion method (Rosco Diagnostica, according to EUCAST methodology), therefore no MIC available. In all determined cases (28/31) the disk diffusion zone diameter was interpreted as resistant (EUCAST Clinical Breakpoint Tables).

**Appendix Table 3.** Whole genome sequencing analysis of the MC0398-MT2306 outbreak strain*

| SSCmec genes | Antimicrobial resistance genes | Virulence genes |
|---|---|---|
| *V(5C2)* | *ant(9)-Ia* (aminoglycoside) | *lukF, lukS* (Panton valentine leucocidin) |
| | *aph(2")-Ia* (aminoglycoside) | *aur* (Aureolysin) |
| | *dfrE* | *hlgA, hlgB, hlgC* (Gamma-hemoysin) |
| | *erm(A)* (streptogramin, olincosamide and macrolide) | *sak* (Staphylokinase) |
| | *erm(B)* (streptogramin, olincosamide and macrolide) | *scn (*Staphylococcal complement inhibitor) |
| | *mecA* (β-lactam) | |
| | *tet(K)* (tetracycline) | |

*Presence of *SSCmec,* virulence and antimicrobial resistance genes in the MC0398-MT2306 outbreak strain, as determined by whole-genome sequencing.
